# Quercetin Protects against Diabetes-Induced Exaggerated Vasoconstriction in Rats: Effect on Low Grade Inflammation

**DOI:** 10.1371/journal.pone.0063784

**Published:** 2013-05-22

**Authors:** Mona F. Mahmoud, Noura A. Hassan, Hany M. El Bassossy, Ahmed Fahmy

**Affiliations:** 1 Department of Pharmacology and Toxicology, Faculty of Pharmacy, Zagazig University, Zagazig, Egypt; 2 Department of Pharmacology and Toxicology, Faculty of Pharmacy, King Abdulaziz University, Jeddah, Kingdom of Saudi Arabia; University of Milan, Italy

## Abstract

Vascular complications are the leading cause of morbidity and mortality in patients with diabetes. Quercetin is an important flavonoid with antioxidant and anti-inflammatory activity. Here, the effect of quercetin on diabetes-induced exaggerated vasoconstriction in insulin deficient and insulin resistant rat models was investigated. Insulin deficiency was induced by streptozotocin while, insulin resistance by fructose. Rats were left 8 weeks or 12 weeks after STZ or fructose administration respectively. Quercetin was daily administered in the last 6 weeks. Then, tail blood pressure (BP) was recorded in conscious animals; concentration-response curves for phenylephrine (PE) and KCl were studied in thoracic aorta rings. Non-fasting blood glucose level, serum insulin level, insulin resistance index, serum tumour necrosis factor-α (TNF-α) and serum C-reactive protein (CRP) were determined. Nuclear transcription factor-κB (NF-κB) was assessed by immunofluorescence technique. Histopathological examination was also performed. The results showed that quercetin protected against diabetes-induced exaggerated vasoconstriction and reduced the elevated blood pressure. In addition, quercetin inhibited diabetes associated adventitial leukocyte infiltration, endothelial pyknosis and increased collagen deposition. These effects were accompanied with reduction in serum level of both TNF-α and CRP and inhibition of aortic NF-κB by quercetin in both models of diabetes. On the other hand, quercetin did not affect glucose level in any of the used diabetic models. This suggests that the protective effect of quercetin is mediated by its anti-inflammatory effect rather than its metabolic effects. In summary, quercetin is potential candidate to prevent diabetic vascular complications in both insulin deficiency and resistance via its inhibitory effect on inflammatory pathways especially NF-κB signaling.

## Introduction

In the last decade the incidence and prevalence of diabetes has risen steeply. Not only the large number of diabetic patients but also the increased mortality and morbidity rates due to increased cardiovascular diabetic complications have got a great attention [1]. While the main goal is prevention of diabetes development and curing the disease, prevention and treatment of complications are equally important [1]. Studies in both animal and human diabetics have shown alteration of several factors that may be fundamental in mediating structural and functional deficits at both the early and the late stages of the disease [2].

There is increasing evidence indicating that there is a strong relationship between inflammatory processes and the development and progression of diabetic complications [3]. Both pro-inflammatory cytokines and adhesion molecules have been proved to be important in the development of diabetic complications [4]. Inflammatory mediators can activate a number of receptors, which subsequently result in β-cell dysfunction, insulin signaling impairment, endothelial dysfunction and altered vascular flow, all of which comprise final pathways that result in the diabetic vascular complications [3]–[6]. Tumour necrosis factor-α (TNF-α) is a pleotropic peptide that plays an important role in several inflammatory and cytotoxic reactions [7]. We have previously reported an important role of TNF-α in the pathogenesis of vascular disease in insulin resistance [8]. TNF-α has been documented to be a powerful mediator of leukocyte recruitment, vascular plasma protein leakage and cellular apoptosis [9], [10]. Moreover, gene transcription for TNF-α is modulated by nuclear transcription factor-κB (NF-κB) [11], which is considered a major intracellular inflammatory mediator of most of the vascular inflammatory response [12]. Furthermore, correlations between C-reactive protein (CRP), a marker of systemic inflammation, and markers of endothelial dysfunction have been reported in both type 1 and type 2 diabetic patients. CRP may act not only as a marker of cardiovascular disease but may also have biological properties that cause endothelial dysfunction. In vitro studies have shown that CRP has a direct proinflammatory effect on human endothelial cells and affects endothelial function [13].

Quercetin (3,3′,4′,5,7 pentahydroxy flavone) belongs to the family of flavonoids, a large class of naturally occurring, low molecular weight plant metabolites [14]. It is found in many foods, including vegetables, tea, fruits and wine [15]. Quercetin is an effective inhibitor of lipoxygenases [16]. It also exhibits a broad range of pharmacological activities such as anti-inflammatory, anti-oxidant [17], anti-tumor, immunomodulatory [18], anti-ulcer [19] and vasodilator [20] effects. It also prevents the oxidation of low density lipoproteins in vitro [21]. Quercetin is considered as peroxyl radical scavenger [22]. Quercetin can also attenuate TNF-α stimulated adhesion molecule expression in human aortic endothelial cells [23].

Previously, some studies indicated that quercetin failed to correct vascular reactivity in spontaneous hypertensive rat models [24]. However, others showed that it has vascular protective effect against diabetes-induced vascular impairment, via preservation of endothelium-derived nitric oxide [20]. But to our knowledge there is no evidence on the role of the anti-inflammatory effect of quercetin on its vasoprotective effect against diabetic vascular complications. So the aim of the present study was to investigate the role of the anti-inflammatory effect of quercetin on the progression of diabetic vascular complications.

## Materials and Methods

### Ethics Statement

Experimental design and animal handling were performed according to the guidelines of the Ethical Committee of the Faculty of Pharmacy, Zagazig University, for Animal Use and in accordance with the recommendations of the Weatherall report. The ethical committee of the Faculty of Pharmacy; Zagazig University approved the study protocol. Every effort was done to minimize the number of animals and their suffering.

### Animals

Adult male albino rats weighing 140–160 g for the insulin deficiency part were used while weighing 90–110 g for insulin resistance part (Zagazig University, Zagazig, Egypt). Rats were housed in clear polypropylene cages (five rats per cage) and kept on a light–dark cycle of equal duration. All rats were kept under constant environmental conditions. Rats were fed commercially available rat normal pellet diet and water ad libitum.

### Study Protocol

Two animals models were used; insulin deficiency and insulin resistance. In each model, animals were randomly divided into four experimental groups (eight animals each); control, quercetin-treated control (Quercetin-control), insulin deficient or resistant, and quercetin-treated insulin deficient (Quercetin-insulin deficient) or resistant (Quercetin-insulin resistance). Insulin resistance was induced first (as this takes 12 weeks) by adding fructose (10%) to everyday drinking water, 4 weeks later insulin deficiency was induced by a single intraperitoneal injection of streptozotocin (STZ, 50 mg/kg). Diabetes was confirmed by a stable hyperglycemia (250-350 mg/dl) after 2 weeks of STZ injection when diabetic rats are divided between groups. Quercetin (50 mg/kg/day) was daily administered as suspension in distilled water by oral gavage in the last 6 weeks of study (which ends in both parts at the same time). Control groups received distilled water as a vehicle. Serum concentration about 5.75 µM is obtained by oral administration of quercetin in a dose of 50 mg/kg as calculated based upon previously published bioavailability study of quercetin in rats [25].

At the end of the study and 12 h after the last injection, blood pressure (BP) was measured and blood glucose level was determined. Blood was collected from the retro-orbital plexus and centrifuged (3000×g, 4°C, 20 min) to separate serum that was divided into aliquots and stored at -20°C till analyzed for insulin level, TNF-α and CRP. Rats were killed by cervical dislocation then, through opening the abdomen, descending thoracic aorta was carefully excised and placed in a Petri dish filled with cold Krebs–Henseleit buffer containing (in mM): NaCl 118.1, KCl 4.69, KH2PO4 1.2, NaHCO3 25.0, glucose 11.7, MgSO4 0.5 and CaCl2 2.5. The aorta was then cleaned of any excess connective tissue and fat and cut into 3 rings of approximately 3 mm in length. For each animal, one aortic ring was suspended in an organ bath for studying vascular reactivity while the other two rings of aorta were fixed in 10% formalin for immunohistochemistry techniques to measure the activation of NF-κB and for histopathological examination to study the level of leukocyte infiltration, pyknosis and collagen synthesis.

### Biochemical Analysis

Blood glucose was determined by glucose meter (Bionime GmBH) that uses noble metal electrode strips. Serum insulin level was assayed by sandwich enzyme-linked immunosorbent assay (ELISA) (Millipore, Cairo, Egypt) which uses microtiter plate coated with mouse monoclonal anti-rat insulin antibodies. The estimated insulin resistance index (HOMA-IR) was calculated using the serum non-fasting glucose and insulin levels according to the following equation [26]: HOMA-IR = glucose concentration (mmol/l) X insulin (µU/l)/22.5. Serum TNF-α level was determined by ELISA using Quantikine® kit (R&D systems, Cairo, Egypt) that contained Escherichia coli-expressed recombinant rat TNF-α and antibodies raised against the recombinant factor. Serum CRP level was assayed using a solid phase ELISA that uses affinity purified anti-rat CRP antibodies for solid phase (microtiter wells) immobilization and horseradish peroxidase (HRP) conjugated anti-rat CRP antibodies for detection.

### Blood Pressure Measurement

Blood pressure (BP) was measured indirectly as described previously [27] in a conscious and slightly restrained rat by the tail cuff method. For these measurements, rats were conditioned for at least 3 days before measurements to the restraint and the warming chamber for10–20 min/day. BP measurements were performed by the same investigator from 7∶00 to 12∶00 AM. After 5–10 min of stabilization in a warming chamber (35°C), a typical run involved 10 repetitions of the automated inflation–deflation cycle. The mean of 6 readings within a 5–10 mmHg range was taken as the BP.

### Vascular Reactivity

Thoracic aorta rings were suspended in individual 30 ml organ chambers containing Krebs–Henseleit buffer at 37°C and aerated with 95% oxygen, 5% carbon dioxide under 1200 mg resting tension. Ring tension was determined by use of an isometric force transducer (Biegestab K30, Hugosachs Elektronik, March, Germany). Force displacement was recorded using a Power Lab Data Interface Module connected to a PC running Chart software (v4.2, ADI Instruments, Chalgrove, Oxon, UK). Rings were equilibrated for 60 min during that time, the bath solution was changed every 30 min. Before beginning the experiment, vessel viability was assessed by exposing arteries to KCl (80 mM). This was repeated until stable responses were achieved (usually two exposures). Cumulative concentrations of phenylephrine (PE, 10-9 to 10-5 M) or KCl (10 to 100 mM) were added to the organ bath and the response was recorded in order to study the contractile responsiveness of aorta.

### Nuclear Transcription Factor-κB (NF-κB) Immunohistochemistry

Immunohistochemistry of NF-κB in rat paraffin embedded aorta sections was assessed using the method described by Szocs et al [28] with some modification. The method uses primary antibody to detect the active form of the NF-κB in sections followed by Rhodamine conjugated secondary antibody. In brief, paraffin embedded slide samples were routinely de-paraffinized, rehydrated and washed in 3% H_2_O_2_ to block endogenous peroxidise activity. Then the slides were incubated successively with gout anti- NF-κB p65 subunit primary antibody (StressGen, Ann Arbor, MI, USA) and Rhodamine conjugated gout anti-mouse IgG secondary antibody (Molecular probes, UK). Images were obtained by LEICA DM500 fluorescence microscope (Leica Microsystems, Wetzlar, Germany) with excitation at 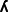 = 550 and emission at 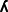 = 570 nm. Images were acquired with identical acquisition parameters, with minimum gain to avoid interference by tissue autofluorescence were done by Image J software. The aorta obtained from each rat was divided between all treatment groups.

### Histopathological Examination

The aorta was rapidly dissected out and tissue sections (5 mm) were fixed by immersion in 10% neutral formalin solution at room temperature. For histological examinations, paraffin-embedded tissue sections of aorta were stained with haematoxylin and eosin (H&E). And for collagen synthesis examination, paraffin-embedded tissue sections of aorta were stained with Collagen-Masson’s Trichrome stain. Sections were examined under light microscope.

### Drugs and Chemicals

The following drugs and chemicals were used: STZ, PE (Sigma-Aldrich, Dorset, UK). Quercetin (Xi’an App-Chem Bio (Tech), Xi’an, China). STZ and quercetin were dissolved in cold distilled water. KCL and PE were dissolved in cold Krebs–Henseleit buffer. All other chemicals were supplied from El-Nasr. Co, Egypt and they are of high purity and analytical grade.

### Statistical Analysis

All data are expressed as mean ± SEM. Statistical analysis was performed by the analysis of variance (ANOVA) followed by Newman-Keuls’ post hoc test. The agonist maximum response (Emax) was calculated from concentration–response curve by non-linear regression analysis of individual curves using computer based fitting program (Prism 5, Graph pad, CA, USA). Values of P<0.05 were considered significant.

## Results

### Biochemical Parameters

Tables 1 and 2 show that oral administration of quercetin (50 mg/kg/day) in the last 6 weeks to normal animals did not affect either body weight, blood glucose, insulin level, HOMA-IR or circulating TNF-α and CRP levels compared with control group.

**Table 1:** Effect of STZ- induced (50 mg.kg^−1^, 8 weeks) diabetes and daily oral administration of quercetin (50 mg.kg^−1^) on body weight, blood glucose, serum insulin, insulin resistance (IR) index, TNF-α, C-reactive protein (CRP), systolic BP.

Parameters	Control	Control-quercetin	Diabetic	Diabetic-quercetin
**Body Weight** (gm)	260.1±8.6	257.5±10.5	278.7±17.0	203.5±19.4##
**Glucose** (mg.dl-1)	120.2±2.8	160.7±17.2	500.1±42.0***	446.9±44.2
**Insulin** (µg.l-1)	15.8±0.5	13.50±0.9	11.2±0.7**	8.9±0.8
**IR index** (units)	4.6±0.2	3.3±0.4	15.4±1.3***	13.2±1.2
**TNF-α** (µg.l^−1^)	73.2±6.1	88.3±8.7	500.1±42.0***	125.1±8.2###
**CRP** (ng.ml^−1^)	10.3±0.4	11.3±0.4	21.5±1.8***	3.8±0.2###
**Systolic BP** (mmHg)	106.8±1.3	113.0±4.7	133.2±1.9***	118.4±4.2###

Values are expressed as the mean ± S.E of mean; N = 8 animals;

* P<0.05,

** P<0.01,

*** P<0.001, compared with the corresponding control group values;

# P<0.05,

## P<0.01,

### P<0.001 compared with the corresponding diabetic group values; by One Way ANOVA and Newman Keuls *post hoc* test.

**Table 2:** Effect of fructose- induced insulin resistance (IR, 10% in drinking water, for 12 weeks) and daily oral administration of quercetin (50 mg.kg^−1^) on body weight, blood glucose, serum insulin, insulin resistance (IR) index, TNF-α, C-reactive protein (CRP), systolic BP.

Parameters	Control	Control-quercetin	IR	IR-quercetin
**Body Weight** (gm)	260.0±7.2	260.8±12.2	363.0±15.5***	364.5±12.2
**Glucose** (mg.dl-1)	115.4±7.1	149.1±18.0	117.6±7.4	110.2±5.7
**Insulin** (µg.l-1)	10.5±0.4	10.6±0.7	17.0±0.9***	4.6±0.9###
**IR index** (units)	3.0±0.3	3.5±0.3	4.8±0.3***	1.2±0.2###
**TNF-α** (µg.l^−1^)	72.1±6.4	92.8±10.6	192.1±10.9***	126.4±6.3###
**CRP** (ng.ml^−1^)	10.4±0.3	11.5±0.5	13.3±0.8**	6.5±0.4###
**Systolic BP** (mmHg)	106.1±3.6	115.4±4.4	129.0±2.2***	118.4±2.9#

Values are expressed as the mean ± S.E of mean; N = 8 animals;

* P<0.05,

** P<0.01,

*** P<0.001, compared with the corresponding control group values;

# P<0.05,

## P<0.01,

### P<0.001 compared with the corresponding diabetic group values; by One Way ANOVA and Newman Keuls *post* hoc test.

Table 1 shows that STZ didn’t affect body weight but led to a sustained (8 weeks) elevation in blood glucose level (P<0.001), significant reduction in insulin serum level (P<0.01) and HOMA-IR (P<0.001) compared to control. Also STZ-diabetic animals had a significantly higher level of serum TNF-α (P<0.001) and CRP (P<0.001) compared to control. Oral administration of quercetin in the last 6 weeks to insulin deficient animals did not affect the developed hyperglycaemia, serum insulin level and HOMA-IR but resulted in significant reduction of body weight (P<0.01), serum level of TNF-α (P<0.001) and serum CRP level (P<0.001) compared to insulin deficient group.

Fructose feeding in drinking water (10% for 12 weeks) caused a significant increase in body weight (P<0.001), serum insulin (P<0.001), HOMA-IR (P<0.01), serum TNF-α level (P<0.001) and serum CRP level (P<0.05) but did not change serum glucose level compared to normal control (Table 2). Oral administration of quercetin in the last 6 weeks reduced serum insulin levels and HOMA-IR (P<0.001) and didn’t affect serum glucose level and body weight. Quercetin administration normalized serum TNF-α level (P<0.001) and serum CRP level (P<0.001).

### Blood Pressure

Table 1 shows that insulin deficiency induced by STZ led to significant elevations in systolic pressure (P<0.001) compared to control group. While quercetin administration abolished the observed elevated systolic (P<0.001).

Fructose induced a significant elevation in systolic (P<0.001) and pressure (P<0.05) compared to control group. While, quercetin administration abolished the observed elevated systolic BP (p<0.05) (Table 2).

Quercetin administration to normal animals did not alter systolic BP compared with control group (Tables 1 and 2).

### Vascular Reactivity

**Figure 1:**
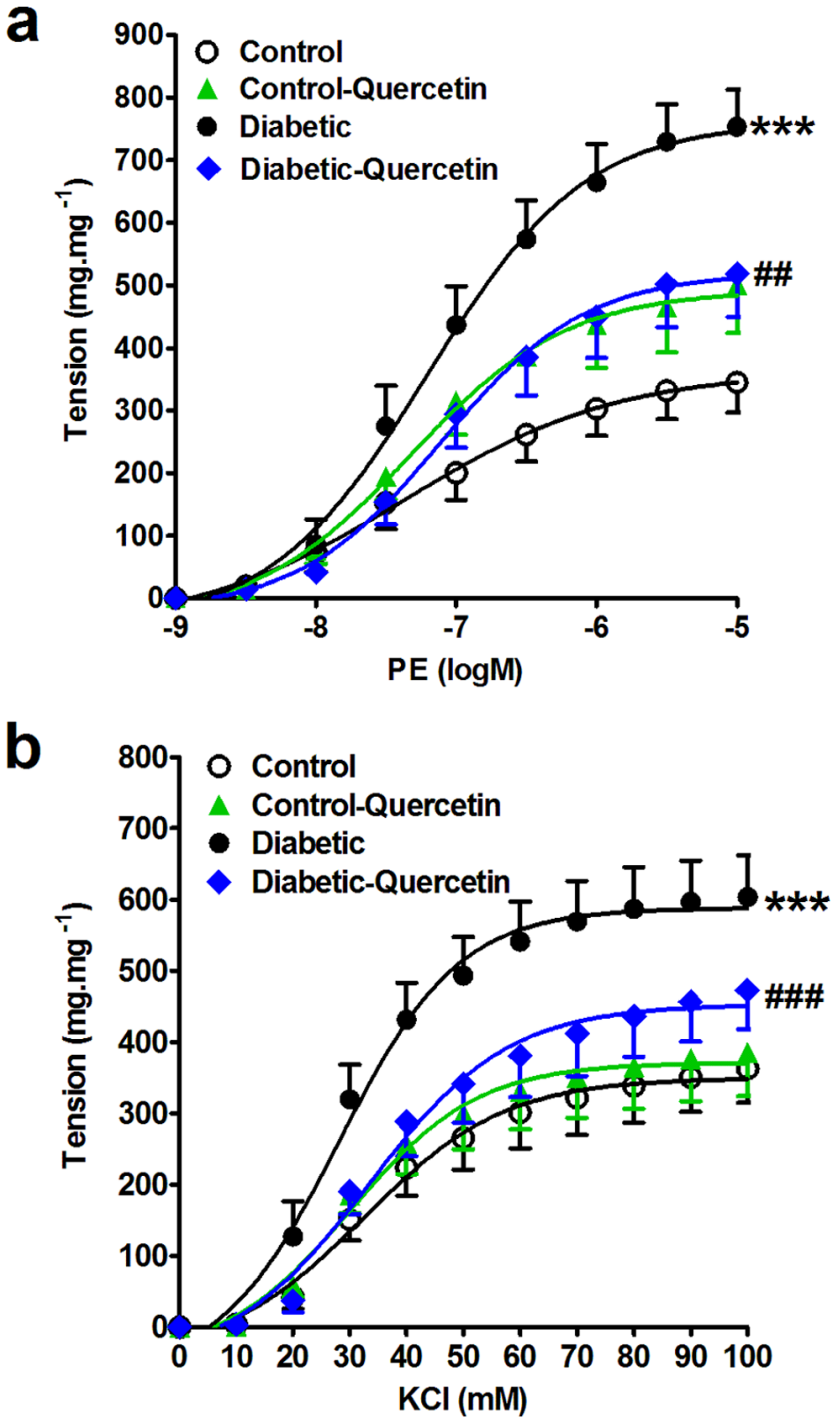
Effect of STZ- induced insulin deficiency (50 mg.kg^−1^, 8 weeks) and daily oral administration of quercetin (50 mg.kg^−1^) on the isolated aorta responsiveness to PE (a) and KCl (b). Symbols indicate mean ± SEM for N = 6–8 animals; ^*^P<0.05, ^**^P<0.01, ^***^P<0.001, compared with the corresponding control group values; ^#^P<0.05, ^##^P<0.01, ^###^P<0.001 compared with the corresponding insulin deficient group values; by One Way ANOVA and Newman Keuls *post hoc* test.

**Figure 2:**
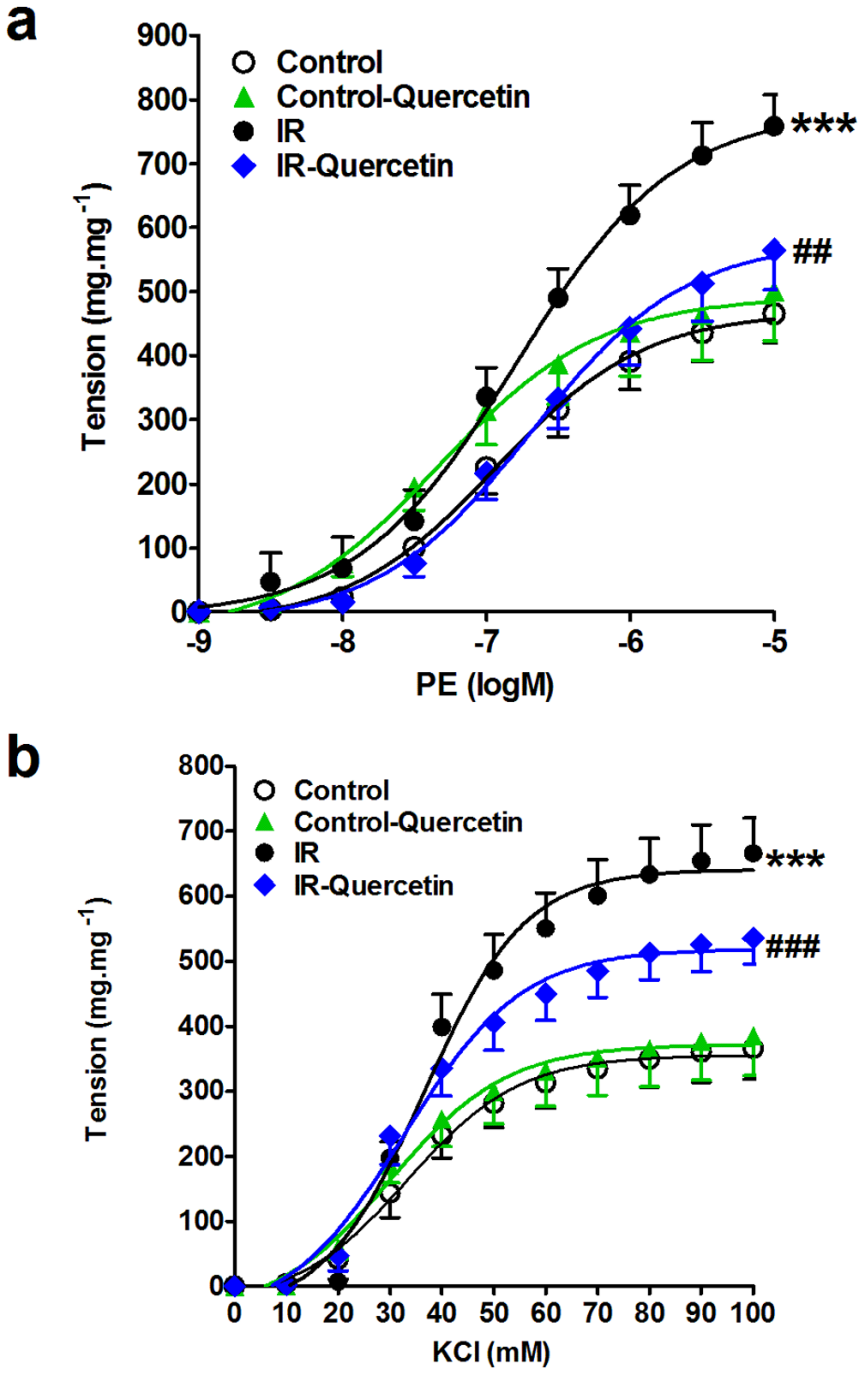
Effect of fructose-induced insulin resistance (10% in drinking water, for 12 weeks) and daily oral administration of quercetin (50 mg.kg^−1^) on the isolated aorta responsiveness to PE (a) and KCl (b). Symbols indicate mean ± SEM for N = 6–8 animals; ^*^P<0.05, ^**^P<0.01, ^***^P<0.001, compared with the corresponding control group values; ^#^P<0.05, ^##^P<0.01, ^###^P<0.001 compared with the corresponding insulin resistant group values; by One Way ANOVA and Newman Keuls*post hoc* test.

Cumulative addition of PE (PE, 10^−9^ to 10^−5^ M) or KCl (10^−2^ to 10^−1^ M) to the organ bath resulted in concentration dependent contraction of aorta in all the groups (Figures 1 and 2).

Figure 1 shows that STZ caused an increase in aorta responsiveness to PE and KCl, reflected by a significant increase in E_max_ (P<0.001). Quercetin administration prevented exaggerated response of aorta to PE (P<0.01) and normalized the aorta response to KCl (P<0.001).

Fructose induced a significant increase in aorta responsiveness to PE and KCl, revealed by a significant increase in E_max_ (P<0.001). Quercetin administration reduced the exaggerated response of aorta to PE (P<0.01) and normalized the exaggerated response to KCl (P<0.001) as shown in Figure 2.

Quercetin administration to normal animals did not significantly affect aorta responsiveness to PE or KCl compared to control group.

### Nuclear Factor Kappa B Immunohistochemistry

**Figure 3:**
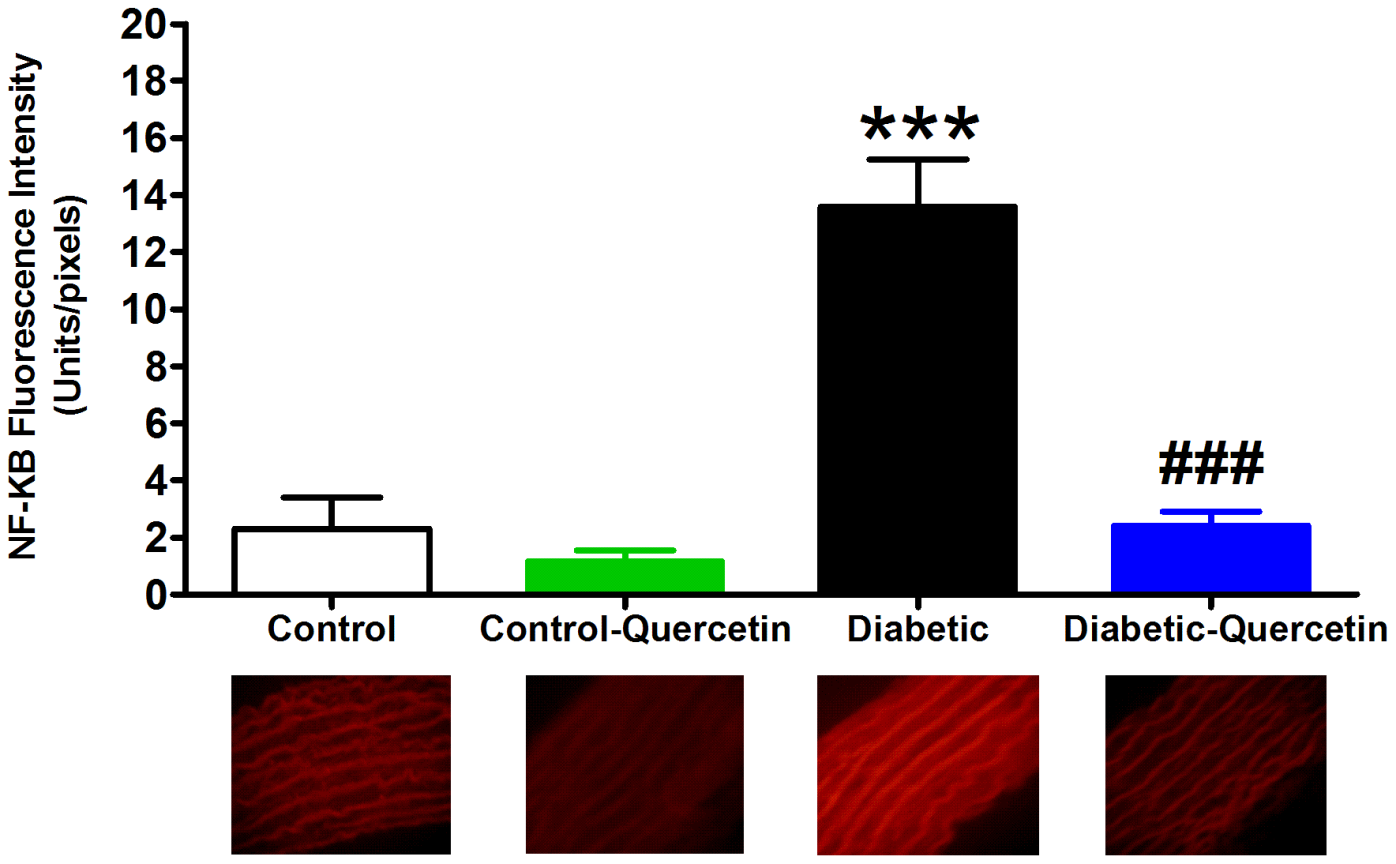
Effect of STZ- induced insulin deficiency (50 mg.kg^−1^, 8 weeks) and daily oral administration of quercetin (50 mg.kg^−1^) on NF-κB activation. Symbols indicate mean ± SEM for N = 6–8 animals; ^*^P<0.05, ^**^P<0.01, ^***^P<0.001, compared with the corresponding control group values; ^#^P<0.05,^##^P<0.01, ^###^P<0.001 compared with the corresponding insulin deficient group values; by One Way ANOVA and Newman Keuls *post hoc* test. Photos at the bottom are representative fluorescence images of aorta cross sections immunofluorescence stained by NF-κB antibody followed by Rhodamine conjugated secondary antibody.

STZ caused an activation of transcription factor NF-κB compared to control group (P<0.0001) as shown in Figure 3. While, quercetin administration prevented the activation of the transcription factor NF-κB (P<0.001) compared to STZ group.

**Figure 4:**
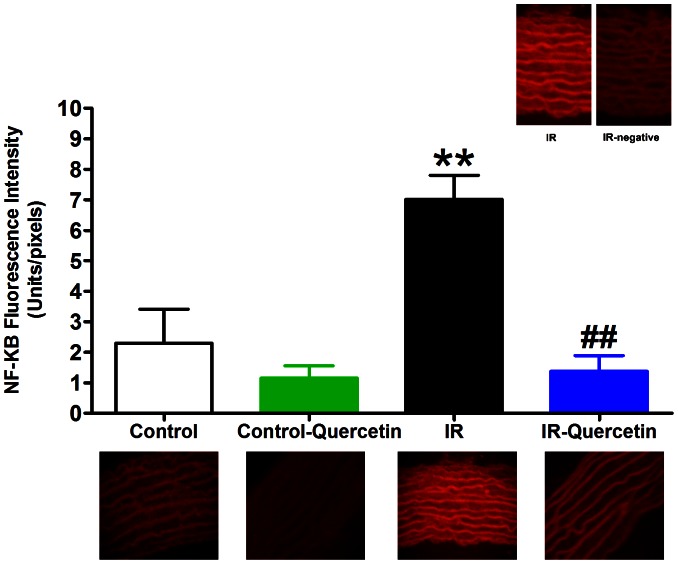
Effect of fructose-induced insulin resistance (10% in drinking water, for 12 weeks) and daily oral administration of quercetin (50 mg.kg^−1^) on NF-κB activation. Symbols indicate mean ± SEM for N = 6–8 animals; ^*^P<0.05, ^**^P<0.01, ^***^P<0.001, compared with the corresponding control group values; ^#^P<0.05, ^##^P<0.01, ^###^P<0.001 compared with the corresponding insulin resistant group values; by One Way ANOVA and Newman Keuls *post hoc* test. Photos at the bottom are representative fluorescence images of aorta cross sections immunofluorescence stained by NF-κB antibody followed by Rhodamine conjugated secondary antibody. Photos at the right top corner are aorta cross section of IR group and its negative control (without the primary antibody).

Fructose induced a significant activation of transcription factor NF-κB compared to control group (P<0.01). While, quercetin administration resulted in a significant reduction in the activation of the transcription factor NF-κB (P<0.01) compared with fructose group (Figure 4).

Quercetin administration to normal animals didn’t affect NF-κB activation compared to control (Figures 3 and 4). Sections treated with the secondary antibody alone did not show specific staining (Figure 4).

### Histopathological Examination

**Figure 5:**
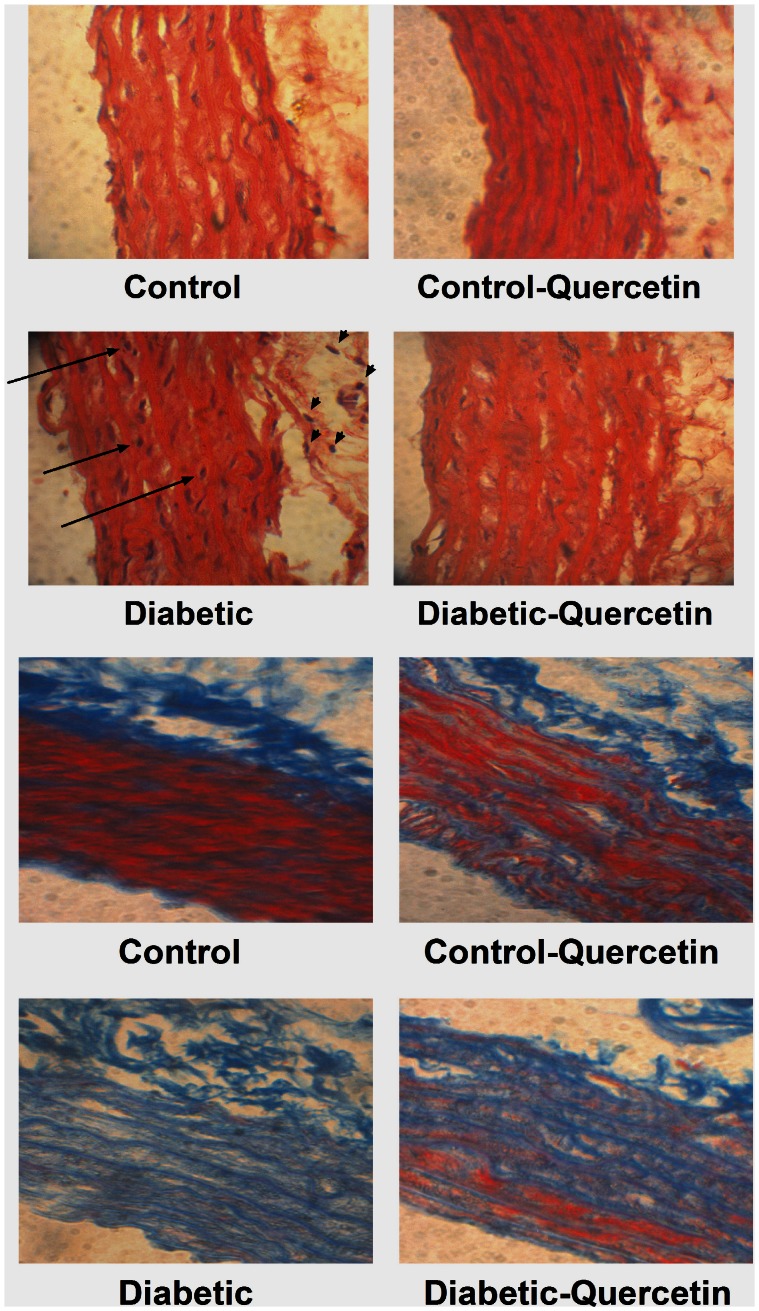
Effect of STZ- induced insulin deficiency (50 mg.kg^−1^, 8 weeks) and daily oral administration of quercetin (50 mg.kg^−1^) on the structure of aorta (a); micrographs are representative of cross sections from 6 rats stained with hematoxylin and eosin (X 1200). Sections are shown with the lumen on the bottom left side of the frames. Arrows show leukocyte infiltration into the adventitia and endothelial pyknosis. And on the level of collagen synthesis (b); micrographs are representative of cross sections from 6 rats stained with Masson’s trichrome (X 1200). Collagen is blue; noncollagen proteins are pink and red.

Figure 5 (a) and (b) show that STZ injection was associated with marked infiltration of leukocytes in the adventitia, pyknosis of endothelial cells and marked increase in collagen deposition (stained by blue color). Quercetin administration prevented the leukocyte infiltration in the adventitia, inhibited the observed endothelial cells pyknosis and resulted in clear reduction in collagen deposition within aorta sections.

**Figure 6:**
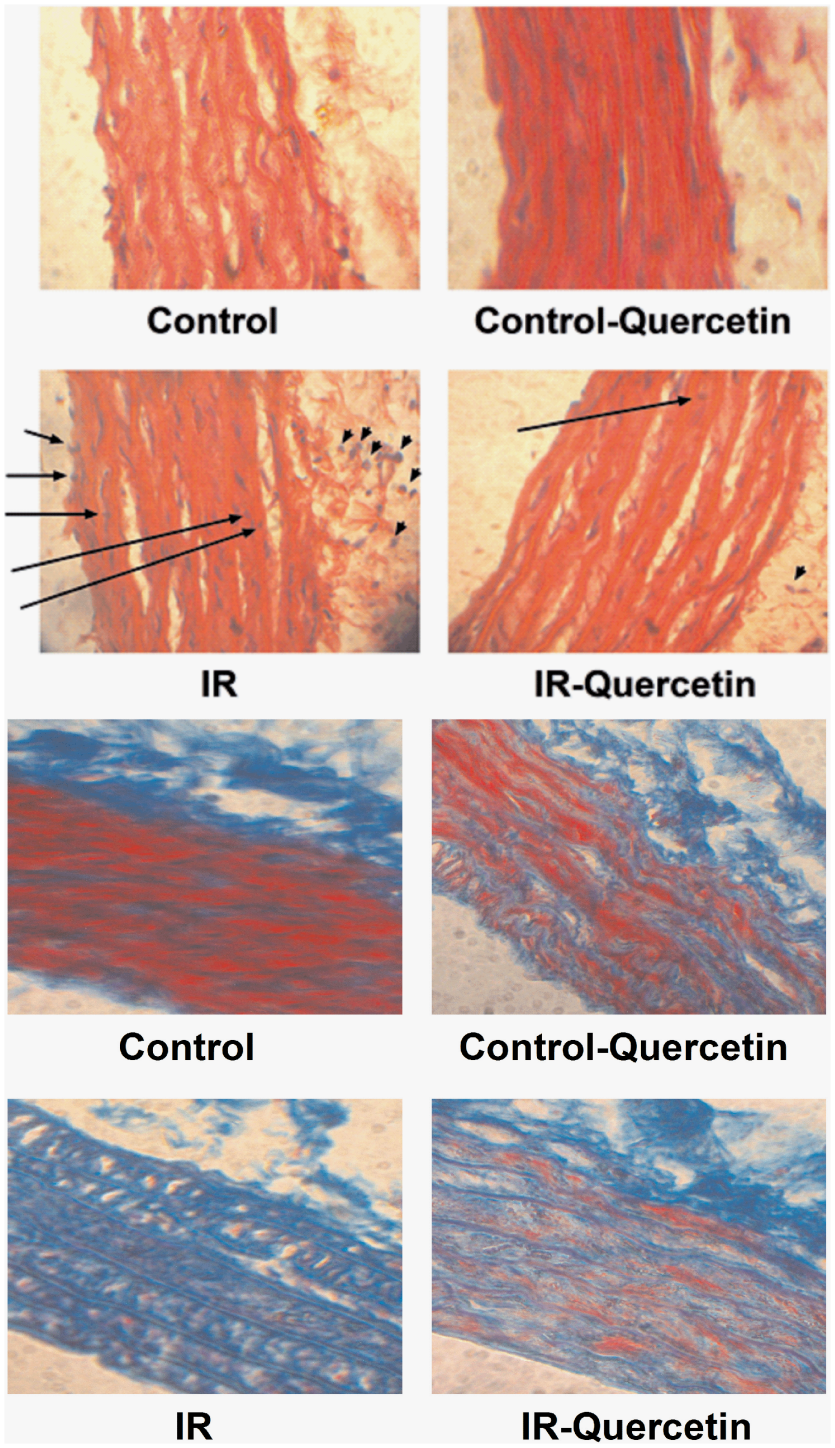
Effect of fructose-induced insulin resistance (10% in drinking water, for 12 weeks) and daily oral administration of quercetin (50 mg.kg^−1^) on the structure of aorta (a); micrographs are representative of cross sections from 6 rats stained with hematoxylin and eosin (X 1200). Sections are shown with the lumen on the bottom left side of the frames. Arrows show leukocyte infiltration into the adventitia and endothelial pyknosis. And on the level of collagen synthesis (b); micrographs are representative of cross sections from 6 rats stained with Masson’s trichrome (X 1200). Collagen is blue; non collagen proteins are pink and red.

Similarly, fructose administration was associated with marked infiltration of leukocytes in the adventitia; pyknosis of endothelial cells and marked increase in collagen deposition (Figure 6 (a) and (b)). QQuercetin administration prevented the leukocyte infiltration in the adventitia, inhibited the observed endothelial cells pyknosis and clearly reduced collagen deposition.

Quercetin administration to normal animals showed normal aorta with no leukocyte infiltration in the adventia or endothelium pyknosis and showed no change in collagen content compared with control animals (Figures 5 and 6).

## Discussion

The purpose of this study was to investigate the role of anti-inflammatory effect of quercetin against diabetes-induced vascular impairment. Our data conclusively showed that treatment with quercetin had the ability to abrogate hypertension progression induced by diabetes together with amelioration of the exaggerated contractile responses of aorta. Moreover, it could also suppress the inflammatory process in aortic tissues in both diabetic models via amelioration of the elevation in TNF-α and CRP serum levels, inhibition of the activation of the transcription factor NF-κB in aorta of diabetic rats and prevention of leukocyte infiltration in the adventitia and pyknosis of the endothelium. In addition, quercetin was able to prevent collagen deposition in aortic tissues in both diabetic models. Finally, it could reduce the developed hyperinsulinemia in fructose fed rats without affecting the hyperglycemia in STZ diabetic rats. As such, the data presents a novel finding for quercetin which possesses a vascular protection effect in two diabetic models.

This vascular protective effect of quercetin was thoroughly examined in two distinct models of diabetes. An insulin deficient model, where STZ has been widely used to produce insulin deficiency via pancreatic β-cell destruction [29], a significant hyperglycemia with hypoinsulinemia were developed within 2 weeks. However, 10% fructose administration in drinking water resulted in significant hyperinsulinemia, and insulin resistance in 6 weeks in the insulin resistant model. Though administration of quercetin did not reduce the developed hyperglycemia in STZ model, it significantly reduced the insulin level and improved the insulin resistance in the fructose model. This finding is in consistence with a previous study showing an improvement in hyperinsulinemia [30].

Being of great importance in the development of hypertension [31] and in particular in diabetes-evoked hypertension [32], functional impairment in vascular reactivity was focused on during this study. Both models, in the current study, showed an increase in the contraction of isolated aorta to PE and KCl. Similarly, it has been reported that diabetes was associated with enhanced aortic response to different vasoconstrictors [27]. This could be attributed to low grade inflammation in the vasculature associated with diabetes. TNF- α,a known inflammatory mediator, has been proved to induce renal vasoconstriction in mice [33]. Furthermore, treatment of aorta sections from normal rats with TNF-α for 1 h was found to enhance vasoconstrictor response to PE [34]. Another inflammatory cytokine, CRP which plays an essential role in vascular inflammation was known to directly decrease the production of the famous vasodilator NO [35]. Upon administration of quercetin, we observed a decreased response in contraction of isolated aorta to PE and KCl in both diabetic models. This amelioration of exaggerated vasopressors response may be attributed to the inhibition of CRP and TNF-α production.

CRP is the most studied inflammatory marker and has emerged as the prototype of inflammatory mediators because of its stability, long plasma half-life, and the absence of circadian variation [36]. In the present work the circulating level of CRP increased in both insulin deficient and insulin resistant animals. It was reported previously that both insulin deficiency [37] and insulin resistance [38] were associated with increased production of CRP. On the other hand, the current study showed that treatment with quercetin reduces the serum level of CRP in both models of diabetes. Furthermore, previous studies demonstrated that quercetin decreases the elevated serum level of CRP during progression and regression of atherosclerosis using hypercholesterolemic diet in rabbits [39].

Rising evidence suggests that TNF-α, a proinflammatory cytokine, possesses a potent proatherogenic effects. TNF-α stimulates leukocyte adhesion to endothelial cells [40] and chemotaxis [41]. In the present study, the circulating levels of TNF-α were increased in both insulin deficient and insulin resistant animals which was consistent with the previous studies [42], [43]. Both hyperglycemia and high fructose diet can directly produce ROS, which, subsequently, have been shown to stimulate TNF-α gene expression [44], [45] via the activation of the redox-sensitive NF-κB, a pleiotropic regulator of many “response-to-injury” genes, such as TNF-α [46]. The current study showed that quercetin attenuates the increase in the serum level of TNF-α in both models of diabetes. This supports the previous findings that quercetin decreases the expression of endogenous TNF-α gene [47].

Nuclear factor-κB is considered as an essential mediator of inflammation [48] via activation of chemotactic cytokines, which play an important role in the recruitment of monocytes/macrophages [49]. The present study showed that NF-κB activation increased in both insulin deficient and insulin resistant animals. Similarly, previous studies have reported that NF-κB activation increases in the two models of diabetes [50], [51]. NF-κB activation might represent a mechanism by which CRP amplifies and perpetuates the inflammatory component of vascular impairment. As CRP was found to induce NF-κB activation in rat vascular smooth muscle cells [52] and bovine aortic endothelial cells [53], treatment with quercetin in this work prevented NF-κB activation in both models of diabetes. Quercetin affects the upstream signaling of NF-κB pathway by inhibiting up regulation of members of the IKK complex, and these effects on the IKK cascade would in turn contribute to inhibition of NF-κB activation [54]. In addition, the effect of quercetin on NF-κB may be mediated through decreasing the phosphorylation state of IκBα and IκBβ which provided a direct mechanism by which quercetin can inhibit the activity of NF-κB, therefore decreasing endogenous TNF-α expression [47] and down-regulating CRP expression [55].

A critical early episode relates vascular inflammation to leukocyte adhesion to endothelial cells, especially by neutrophils which are considered as the first immune cells to arrive at inflammatory sites [56]. Therefore, in order to substantiate our inflammation data, the present study next examined the aorta sections histopathologically to reveal a marked leukocyte infiltration in the adventitia and endothelial cells pyknosis in both models taken in consideration what reported in the previous studies [57], [58] where both insulin deficiency and insulin resistance had the same results. Treatment with quercetin resulted in inhibition of leukocyte infiltration and protection of endothelial cells against pyknosis as well. Previous reports demonstrated that quercetin inhibited leukocyte infiltration and protected against structural changes in the kidney of rats in response to lead- induced oxidative stress [59]. Importantly, we confirmed that the protective effect of quercetin against leukocyte infiltration and pyknosis is attributed to its anti-inflammatory effect thus inhibiting gene expression of adhesion molecules and chemotaxis.

Collagen is the major biomechanical component of the vessel wall, fibrous collagen during pathological modifications might change the wall stiffness and consequently derange vascular function [60]. In order to further translate this study, the synthesis of collagen was seen to be increased in the diabetic aortas compared to controls in both insulin deficient and insulin resistant animals. Increased collagen level was found in the descending aorta sections from diabetic rats [60] and in the intimal and medial layers of the internal mammary arteries from dyslipidemic, diabetic pigs [61]. Mechanical stretch was found to enhance collagen synthesis in a cultured smooth muscle cells or whole artery segments and this provides a link between local hemodynamic forces and plaque collagen production [62]. This enhancement in turn decreases arterial distensibility and enhances vessel stiffness. It has been mentioned that the modifications in the arterial wall viscoelastic properties increase vascular stiffness, contribute to systolic arterial pressure and BP increment, and have a modified response to vasoactive stimuli [63]. Besides that, insulin per se was found to augment collagen synthesis in the vascular wall of an insulin resistant animal [64]. Interestingly, treatment with quercetin resulted in a significant reduction in collagen synthesis in both diabetic models. This result confirms what was mentioned previously where quercetin had the ability to reduce collagen deposition in the neointima of rat’s abdominal aortas [65]. Thus, we can reveal that the anti-inflammatory effect of quercetin indirectly reduced collagen deposition within aorta sections through reducing mechanical stretch in both models. Moreover, the antihyperinsulinemic effect of quercetin also contributes to preventing collagen deposition in insulin resistant model.

It was reported previously that fructose enriched diet induces an increase in BP in rats [66]. Studies have demonstrated the potential of hyperglycemia in developing hypertension and related vascular disorders in diabetic rats [67]. Therefore taken with this data we showed here that diabetes is accompanied with elevation in systolic BP in both insulin deficiency and insulin resistance models which is in accordance to other studies in insulin deficiency and insulin resistance models respectively [68], [27]respectivey. The increased systolic BP could be secondary to an increased after load or reduced myocardial contractility in diabetic subjects [69] and is an indirect index of arterial stiffness [70]. The present study revealed that quercetin administration abrogated the elevation of systolic BP in both diabetic models. It was reported previously that quercetin reduces BP in adult spontaneously hypertensive rats [71]. It has also been suggested that its ability to reduce BP in animal models of hypertension could be partly explained by direct vasodilator effects [72] which seems to be due to reducing the level of TNF-α, as blockade of TNF-α was found to normalize BP in model of angiotensin II- induced hypertension [73]. Thus the vascular protective effect of quercetin administration in insulin deficiency group obviously was due to direct anti-inflammatory effect of quercetin armed with the knowledge that it did not affect glucose level at the used dose and duration of administration. While in insulin resistance model it appears that both improvements in insulin resistance and a direct anti-inflammatory effect of quercetin corporate to produce this protection.

The effect of administration of quercetin on normal rats was examined to determine whether if any of the exhibited pharmacological effects of quercetin was due to toxic effects of quercetin. The present study showed that oral administration of quercetin (50 mg/kg) to normal rats for six weeks didn’t affect any of the measured parameters compared to the control group thus the pharmacological effects exhibited by quercetin are not due to the toxic effects of the selected dose and duration of administration.

### Conclusion

In conclusion, quercetin offsets the hypertensive effects of diabetes via ameliorating significant vascular functional and structural derangements caused by diabetes in insulin deficiency model due to its direct anti-inflammatory effect and in insulin resistance model due to direct anti-inflammatory effect and indirectly through inhibition of insulin resistance.
